# The impact of the COVID-19 pandemic on the national HPV immunization program in Malaysia

**DOI:** 10.3389/fpubh.2022.907720

**Published:** 2022-08-01

**Authors:** Sharvina Ramesh Rao, Nirmala Kampan, Kah Teik Chew, Mohamad Nasir Shafiee

**Affiliations:** Department of Obstetrics and Gynaecology, Faculty of Medicine, UKM Medical Centre, Kuala Lumpur, Malaysia

**Keywords:** HPV vaccine, COVID-19, cervical cancer, immunization, vaccine

## Abstract

In Malaysia, the HPV immunization program has been introduced since 2010 as part of the national immunization plan for female students aged 13 years old. It was a very successful immunization program with good responses from students and parents until the start of COVID-19 pandemic in 2020. The COVID-19 pandemic caused the schools to be closed and resulted about 225000 female students aged 13 years old either missed their vaccination or have incomplete doses of HPV vaccination in 2020 and 2021. This could possibly lead to an increase in cases of cervical cancer and genital warts in the upcoming years. Hence, a wellorganized catch-up HPV vaccination program is vital in ensuring the aim of achieving zero HPV-related infections in the future.

## Introduction

The Human Papillomavirus (HPV) vaccination was introduced by the World Health Organization (WHO) in 2006. Countless countries have integrated this vaccination into their national vaccination program in their effort to make HPV vaccination their primary strategy in the prevention and eradication of cervical cancer. Since then, three vaccines has been approved for global use by WHO, which are bivalent HPV-16/18 vaccine (Cervarix^®^), quadrivalent HPV-6/11/16/18 vaccine (Gardasil^®^) and nanovalent HPV-6/11/16/18/31/33/45/52/58 (Gardasil^®^). WHO has set a global goal to eliminate cervical cancer by 2030, and one of the targets is for 90 percent of girls aged 9 to 15 to be fully vaccinated against HPV (1).

## HPV immunization program in Malaysia

Since the introduction of HPV vaccine in 2006, Malaysia had inter-agencies and multi-sectoral collaborations to introduce this vaccination in the Malaysian national immunization program. As the result, the National HPV Immunization Program was introduced in August 2010. The National HPV Immunization Program was led by Ministry of Health (MOH) with help from Ministry of Education (MOE), focused on vaccinating school girls aged 13 years old through school-based health service package. The school-based health package was designed to covered school-based health screening and services for students from primary school Standard 1 (age 7) till secondary school From 3 (age 15) (Table 1). The HPV vaccine was given to school girls aged 13 during secondary school From 1. The vaccine was delivered *via* intramuscular injection of 3 doses of quadrivalent HPV vaccine follow the dosing schedule of 0, 2, and 6 months interval. After 2015, following the new recommendation by WHO, the standard dosing schedule has been changed to 0 and 6 months interval for school girls below 15 years old (2, 3).

**Table 1:** School-based health package provided by MOH Malaysia.

**Grade and age**	**Services provided**	**Immunization program**
Standard 1 (age 7)	Body mass index monitoring, visual and hearing screening, cardiovascular and skeletal examination, learning disability detection	BCG, dipheria, pertussis, polio, mumps, measles, and rubella
Standard 6 (age 12)	Body mass index monitoring, visual and hearing screening, cardiovascular and skeletal examination, color blindness screening	None
Form 1 (age 13)	None	HPV vaccine for girls only
Form 3 (age 15)	Body mass index monitoring, visual screening, cardiovascular and skeletal examination, adolescent health education	Tetanus booster

*Family Health Department, Ministry of Health Malaysia*.

To date, the national HPV Immunization Program has been implemented in 2,960 schools registered under MOE in urban and rural areas (4). To ensure all school girls received complete HPV vaccine doses, health care workers (nurses, doctors and medical paramedics) were mobilized from health clinics under MOH to support the vaccination program. A special task force spearheaded by director of Family Health Development Division, was formed to monitor the progress and adverse event of the vaccination program (5). Campaign, seminars and talks in regards of HPV vaccine were conducted through mass media and social media to cultivate the awareness of HPV vaccination as primary prevention for cervical cancer in the public. As the result, there was significant increment of awareness, knowledge and acceptance of HPV vaccination among public through several studies conducted locally (6, 7).

## Status of HPV immunization program before COVID-19

Among the challenges faced during the initial phase (2010–2011) was the lack of parental consent for their children to be given HPV vaccination. Two main contributing factors were identified: (1) concern of ingredients used to manufacture HPV vaccine and (2) poor knowledge of HPV vaccine against cervical cancer prevention (6, 8). A local study found that only 8.6% of parents/guardians knew that the ingredients used to manufacture HPV vaccine met Islamic requirements (8). In 2010, only 95.9% of parents/guardians consented for their children to be given HPV vaccine (9). It was estimated only 55% of young female adults had the intention to receive HPV vaccination (6). Nevertheless, MOH had collaborated well with national Islamic religious authority to provide a comprehensive guideline regarding the usage of HPV vaccine for the best interest of protecting women against cervical malignancy. The guideline was widely used to brief teachers, parents/guardians during seminars or roadshow.

During the sustained implementation phase (2012–2019), the national HPV immunization program was very successful. The HPV Immunization Program aim to vaccinate about 250,000 school girls aged 13 years old every year. It was observed that about 98% of parents/guardians consented for their children to be given HPV vaccine from 2012 to 2016 (9). During the same period, more than 99% of school girls with parental/guardians consent completed full dose of HPV vaccination (9). National statistic from MOH revealed that 85.8% (85.3–91.1%) of school girls aged 13 years old received complete dose of HPV vaccine from 2010–2016 (10).

## Impact of pandemic toward global HPV immunization program

Globally, the pandemic has left a huge impact in multiple sectors. According to WHO and the United Nations Children's Fund (UNICEF), the COVID-19 pandemic has caused 23 million children and 1.6 million girls globally to miss out on scheduled vaccinations and HPV vaccinations respectively in 2020 (11). In Malaysia, the COVID-19 pandemic forced MOE to close the schools and opted for online learning for almost 2 years. In 2020, both primary and secondary schools were closed from March to December. In 2021, schools reopened back for physical teaching and learning in stages, with priority given to students in higher grade (Form 4 and Form 5, aged 16–17 years old) for preparation in national examination. Fortunately, all schools has been back to physical classes since March 2022. This resulted in major disruption and discontinuity of national HPV Immunization Program. MOH estimated 225,000 school girls aged 13 years old would have either missed their vaccination or have incomplete doses of HPV vaccination in 2020 and 2021. Based on the MOE statistics, the total enrolment of female students into Form 1 (aged 13 years old) of government secondary schools was 206,229 students in 2020 and 217,475 students in 2021 (12). By using the mean coverage of 85.8% school girls aged 13 years old get HPV vaccination, mathematic projection revealed that about 176,944 and 186,593 school girls might miss the vaccine through the national HPV vaccination program in 2020 and 2021 respectively. The national HPV Immunization Program is the only option for teenage girls aged 13 years old to be vaccinated with free HPV vaccine. Unfortunately, due to the constraint of health care workers, MOH was unable to provide additional HPV vaccination program for school girls at government healthcare clinics from 2020 to 2021. Although private clinics and hospitals provided the HPV vaccination, parents were not ready to pay for extra cost of the HPV vaccine for their children.

The economic impact caused by the COVID-19 pandemic had forced government to redistribute the national budget to strengthen the economic recovery rather than improving cervical cancer screening program. The total cost of the national HPV Immunization Program was estimated to be RM322.2 million where the cost of purchasing HPV vaccine was RM 319.2 million and the remaining RM 3 million was used in covering aspects of health education, training, and logistics of the involved personnel (4). It also showed that the estimated cost per person would be around RM1000 to RM 1200. During the COVID-19 pandemic, priority has been given to purchase the COVID-19 vaccine to curb the disease. Schools have been utilized for COVID-19 vaccination center. This would have disrupted the cold chain storage system of the HPV vaccination which inevitably increased total cost of the national HPV Immunization Program and eventually lead to an increase in expenses in the healthcare sector by the government. In addition to that, COVID-19 has also reduced vaccine access and disrupted vaccine administration services. A study reported that the impact of the pandemic caused the shortage of personal protective equipment leading to fear and increased anxiety among healthcare workers (13). The study highlighted the disruption to the global trade and supply chains leading to the shortage of HPV vaccination caused by the COVID-19 pandemic (13).

## Discussion

### Long term impact of missed vaccination

The long-term void caused by the HPV immunization program would possibly lead to an increase in cases of cervical cancer and genital warts in the upcoming years. A model-based analysis conducted in the United States revealed that the HPV vaccine coverage reached as low as 23% of the previous year's rate during the COVID-19 pandemic in 2020. This resulted in 51% cases of genital warts, 59% CIN 1 cases, and 55% CIN 2/3 of cases were projected to occur within the first 25 years in the current status quo of dropped vaccination rates due to the COVID-19 pandemic (14). Under the least optimistic assumption of this model-based analysis, there will be over 16,900 additional cases of genital warts, 40,000 cases of CIN 1, and 92,000 cases of CIN 2/3 over the next 50 years (14). This model-based analysis has clearly demonstrated the impact of the disruption caused by the COVID-19 pandemic where it would slow down the tremendous progress of the HPV vaccination rates achieved globally besides increasing the incidence of cervical cancer in the future. MOH projected about 225,000 school girls aged 13 years old would have either missed or have incomplete doses of HPV vaccination during the 2 years duration. In Malaysia, modal based analysis projected that HPV Immunization Program alone would be able to prevent 27,000–32,200 cases of cervical cancer by the year 2070 (15). Thus, active measures such as a catch-up HPV vaccination program should be implemented in Malaysia and globally.

### Perspective of the catch-up program

As the national HPV immunization program has been disrupted by the pandemic, there is a need to put in place catch-up vaccination programs to ensure the closure of the large gaps created by COVID-19. A model-based study conducted in Japan showed that over a 100-year time horizon, there will be earlier and greater reductions in cervical cancer incidence through catch-up HPV vaccination programs where up to 484,248 cases of cervical cancer will be avoided over a 100-year span from 2021 to 2121 (16). Furthermore, this study also showed that there would be a significant reduction of HPV-related diseases such as anogenital warts where an additional 364,721 cases could be prevented by continuing the routine HPV vaccination in both men and women (16). The modal based was calculated with presumption of 42% HPV vaccine coverage among young women. Thus, early measures such as HPV catch-up vaccinations must be set in place. The biggest question here is “what are the challenges that will be faced by the introduction and implementation of a catch-up HPV vaccination program in Malaysia?”

There are many aspects that need to be taken into account before the implementation of this program. From the parents' and individual point of view, they would query about the presence of interaction between HPV vaccination and other vaccinations particularly COVID-19 vaccination. There will be possibilities of vaccine hesitancy because they will be worried about the severity of side effects and adverse effects from the interaction between the HPV vaccination and COVID-19 vaccination. Besides that, the school administration plays a huge role in the implementation of this program. Before the implementation of the HPV catch-up program, the willingness of the schools to allocate time in the midst of catching up back on the missed curriculum that was caused by the multiple lockdowns carried out nationwide needs to be thoroughly assessed. The school will also need to arrange and limit the number of students taking the vaccination daily in line with the adherence to the rules and regulations put in place for the COVID-19 pandemic. The school's administration needs to set up with a system to successfully implement the catch-up HPV immunization program in their respective schools. This will surely pose a great challenge to the teachers to effectively arrange the timetable to give space for the students to receive their HPV vaccination. There should be collaborative efforts between MOH and MOE to effectively carry out the administration of HPV vaccination. MOH will need to provide adequate personal protective equipment and allocate healthcare workers to be sent to schools to carry out the administration of HPV vaccinations. MOH could also use this opportunity to overcome the lack of awareness among the public regarding the importance of HPV vaccination. The current pandemic will provide potential fertile grounds for overcoming doubts, suspicious, rumors, and conspiracy theories regarding vaccination in general among the members of the community (17). In conclusion, the catch-up HPV vaccination program is vital in ensuring the aim of achieving zero HPV-related diseases in the future in Malaysia.

## Author contributions

SR contributed to initial drafting. NK and CT performed first revision. MS completed the final revision of the manuscript. All authors contributed to the article and approved the submitted version.

## Conflict of interest

The authors declare that the research was conducted in the absence of any commercial or financial relationships that could be construed as a potential conflict of interest.

## Publisher's note

All claims expressed in this article are solely those of the authors and do not necessarily represent those of their affiliated organizations, or those of the publisher, the editors and the reviewers. Any product that may be evaluated in this article, or claim that may be made by its manufacturer, is not guaranteed or endorsed by the publisher.
